# Refractory Status Epilepticus Treated With Bilateral Pulvinar Deep Brain Stimulation—A Case Study

**DOI:** 10.1002/acn3.70268

**Published:** 2025-12-04

**Authors:** Mengxuan Tang, Amerta Bai, Felipe Rodridgues Marques Ferreira, Sandipan Pati, Thaddeus Walczak, Benjamin Miller, Oladi Bentho, Thomas Henry, Ilo Leppik, Minoo Shams, Zhiyi Sha, Zachary Sanger, Theoden I. Netoff, Thomas Lisko, Anant Naik, Robert McGovern, Sima Patel

**Affiliations:** ^1^ Department of Neurology University of Minnesota Medical Center Minneapolis Minnesota USA; ^2^ Department of Biomedical Engineering University of Minnesota Minneapolis Minnesota USA; ^3^ Department of Neurosurgery University of Minnesota Medical Center Minneapolis Minnesota USA

**Keywords:** deep brain stimulation, epilepsy, status epilepticus, thalamic neuromodulatory targets

## Abstract

New‐onset refractory status epilepticus (NORSE) arises without an identifiable cause or prior epilepsy history, with a 16%–27% mortality rate and significant long‐term neurological sequelae. Neuromodulation such as deep brain stimulation (DBS) targeting the anterior and centromedian thalamic nuclei has shown promise when the traditional approach of anti‐seizure medications (ASMs), anesthetics, and immunomodulation fails. We present a case of cryptogenic NORSE in a 30‐year‐old male with autism and developmental delay, with refractory seizures localized to bilateral posterior quadrants. Sensing‐enabled DBS targeting the pulvinar thalami led to decreased seizure burden and clinical improvement, highlighting the importance of tailoring neuromodulatory targets to seizure localization.

## 1 Introduction

New‐onset refractory status epilepticus (NORSE) presents as status epilepticus without an identifiable cause, including structural, toxic, or metabolic factors, and without a prior history of epilepsy or other neurological disorders [1]. NORSE carries a mortality rate of 16%–27% in adults and often results in significant long‐term neurological impairment [2, 3, 4]. FIRES is a subcategory of NORSE characterized by a febrile illness occurring 2 weeks to 24 h prior to the onset of refractory status epilepticus. The international consensus for managing NORSE and FIRES includes anti‐seizure medications (ASMs), anesthetics, antivirals, and immunomodulatory therapies [5]. When these methods prove ineffective, emerging neuromodulation techniques such as deep brain stimulation (DBS), responsive neurostimulation (RNS), and electroconvulsive therapy (ECT) may be considered [6]. The thalamus is a recognized target for neuromodulation in refractory epilepsy, demonstrating effective seizure reduction in case series with super‐refractory status epilepticus [7], with most reports focusing on anterior nucleus (ANT) and centromedian nucleus (CMT) [8, 9, 10, 11, 12, 13].

We present a case of cryptogenic NORSE with seizure activity localized to the bilateral posterior quadrants. Based on this localization, the patient underwent bilateral pulvinar DBS implantation, resulting in improvements in seizure burden and clinical exam. This case explores pulvinar as a new target for neuromodulation in the acute management of super‐refractory status epilepticus when traditional management strategies prove insufficient.

## 2 Case Description

A 30‐year‐old male with autism and developmental delay presented with multiple witnessed bilateral tonic–clonic seizures following fever and an upper respiratory illness, which subsequently progressed to super refractory status epilepticus. At baseline, he was ambulatory, minimally verbal, and his functional status had been stable for many years with no reported history of prior seizures. MRI brain demonstrated transient diffusion restriction in the bilateral striatum, hippocampi, and splenium of the corpus callosum. The etiology of the NORSE was suspected to be autoimmune as the CSF was positive for oligoclonal bands, 14‐3‐3, tau, and soluble IL‐2r. Extensive infectious, inflammatory, and genetic workup was otherwise unremarkable, including negative metagenomic next‐gen sequencing from the CSF, autoimmune encephalitis panels from the CSF and serum and whole‐exome sequencing with copy number variation analysis (Table S1).

Multiple EEG patterns on the ictal‐interictal continuum were observed over 4 months of continuous video EEG monitoring, including generalized periodic discharges at frequencies of up to 3 Hz with shifting maxima, in addition to up to 10–20 focal electroclinical seizures per hour arising independently from both hemispheres, most frequently originating in the bilateral posterior regions. Clinically, seizures were occasionally associated with altered awareness, eyelid fluttering, and lip‐smacking behaviors.

**FIGURE 1:**
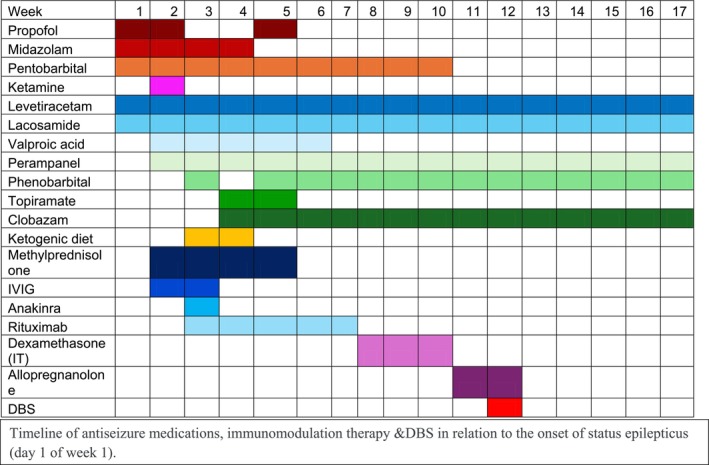
Timeline of antiseizure medication and immunomodulation therapy in relation to the onset of status epilepticus (Day 1 of Week 1).

The patient was empirically treated with intravenous immunoglobulins (IVIG), anakinra (recombinant IL‐1 receptor antagonist), methylprednisolone, rituximab, and intrathecal dexamethasone (Figure 1). His seizures proved refractory to multiple ASMs including levetiracetam, lacosamide, valproate, propofol, midazolam, ketamine, topiramate, perampanel, clobazam and ultimately required prolonged pentobarbital coma for seizure suppression, with consistent re‐emergence of focal nonconvulsive status epilepticus during multiple weaning attempts. Phenobarbital was eventually added to facilitate pentobarbital weaning, and levels were uptitrated to 120–150 μg/mL due to reported efficacy and tolerability at such high doses in super refractory status epilepticus [14]. Five days prior to DBS implantation, allopregnanolone was given under an emergency investigational new drug (EIND) authorization due to its reported tolerability and utility in weaning off third‐line agents in super‐refractory status epilepticus [15]. Seizures were suppressed for the duration of allopregnanolone administration but resumed within 48 h of infusion cessation.

## 3 Results

**FIGURE 2:**
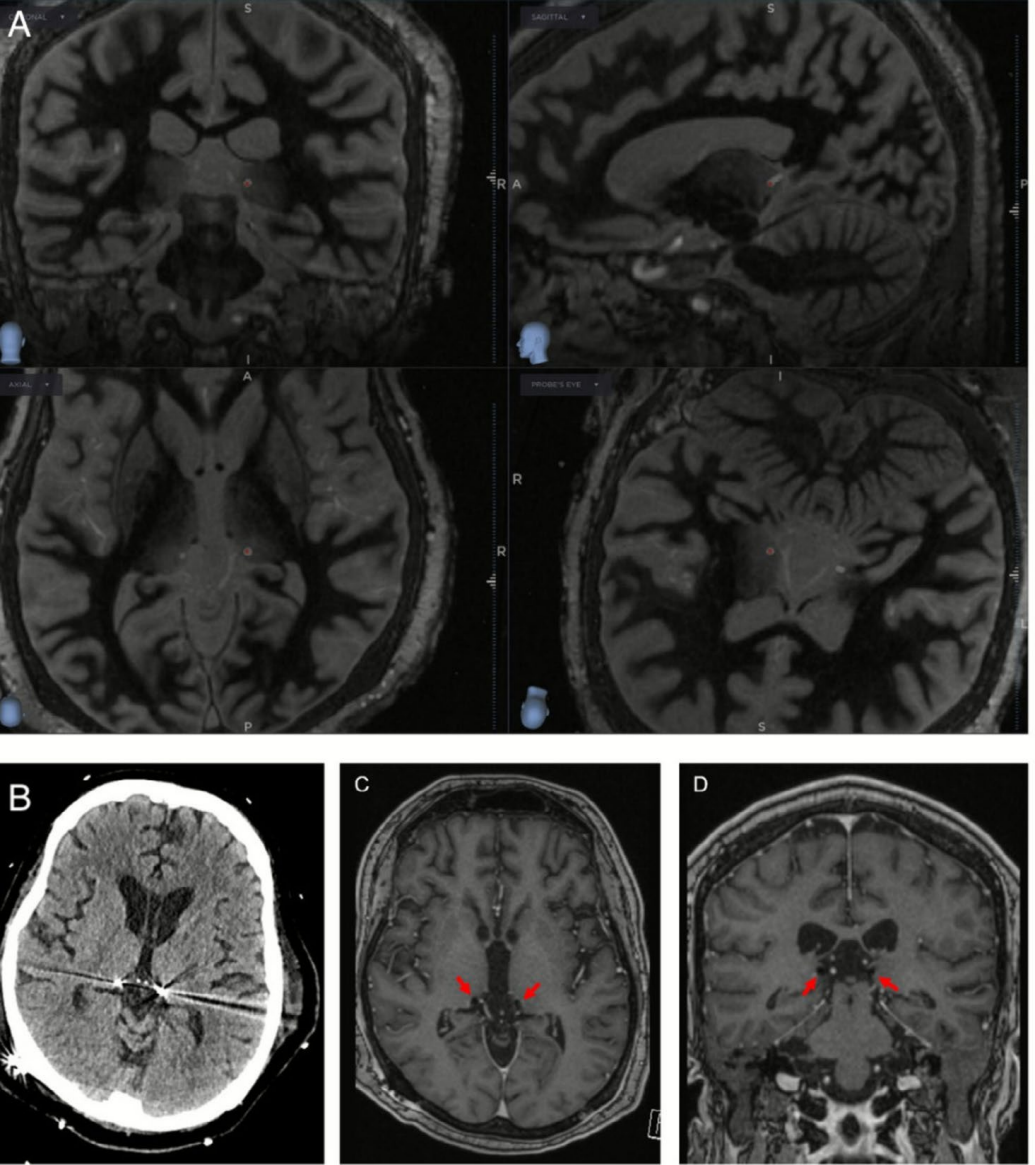
Intraoperative 3Tesla MRI brain with Fast Gray Matter Acquisition T1 Inversion Recovery (FGATIR) sequences documenting DBS lead placement (A). Follow up CT brain on day one (B) and MRI brain 2 months (C and D) post‐op.

After extensive multidisciplinary discussions involving Neurosurgery, Epilepsy, and Neurocritical Care teams, the patient underwent bilateral pulvinar thalamic DBS placement on hospital day 66. Bilateral Medtronic SenSight DBS electrodes were placed with the assistance of intraoperative MRI guidance (Clearpoint Neuro, Solana Beach, CA), which enabled direct visualization of pulvinar nuclei bilaterally using FGATIR sequencing (Figure 2). The stereotactic coordinates were as follows in reference to the mid‐commissural point as defined in the AC/PC coordinate system: L pulvinar nucleus: *X* = −11.2, *Y* = −18.7, *Z* = +4.0, R pulvinar nucleus: *X* = 13.6, *Y* = −17.2, *Z* = +2.2. The case was completed with 0.1 mm radial error on both sides.

**FIGURE 3:**
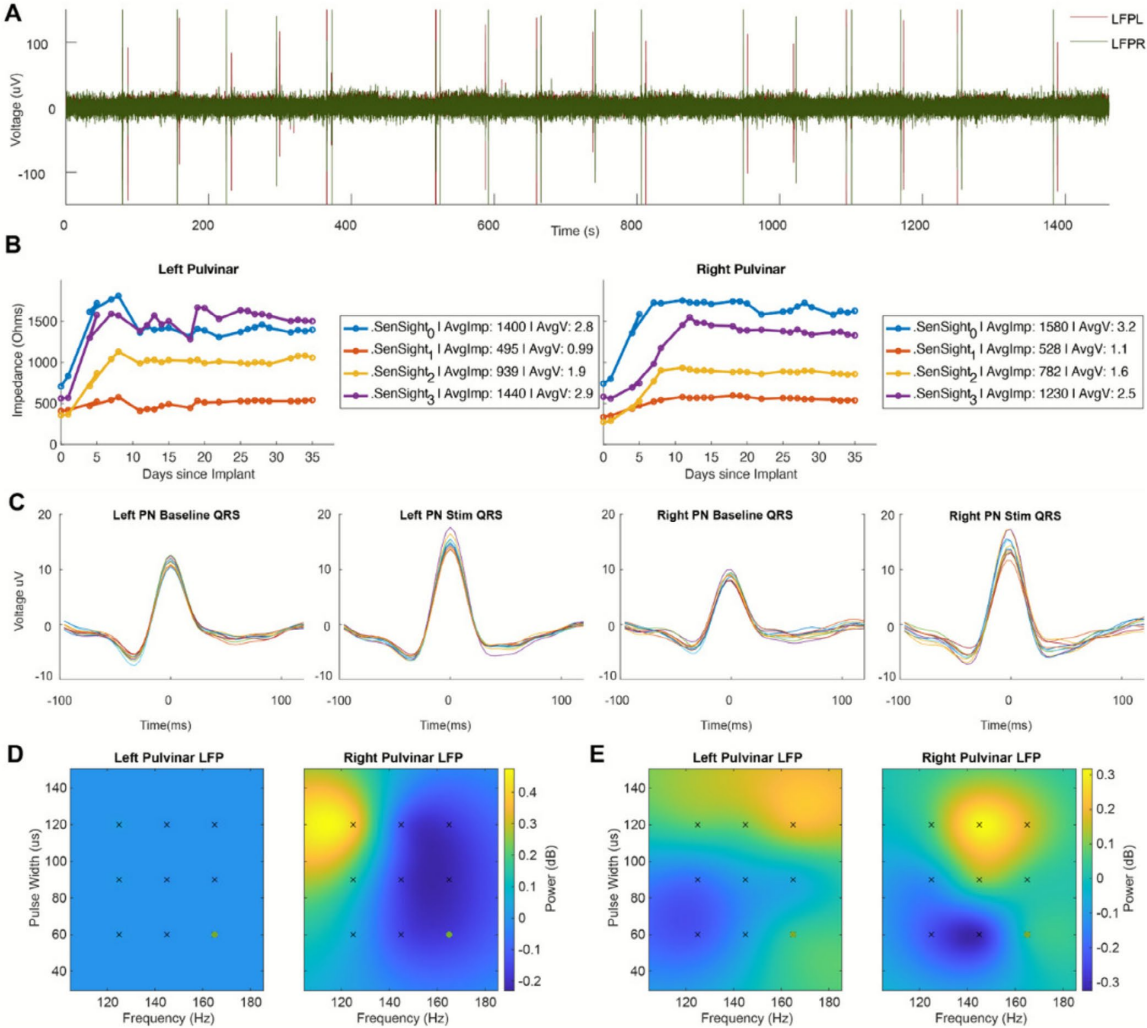
Local Field Potential Response to Pulvinar Deep Brain Stimulation. (A) Raw left and right pulvinar nucleus local field potential (LFP) response recorded from ring contacts 0 and 3 during stim off and stim on of 9 different stimulation settings. (B) Medtronic SenSight electrode contact ring impedance changes over time since implantation in the left and right pulvinar nucleus. The legend for each hemisphere shows the average impedance over time in ohms and the average voltage applied if you assumed a constant 2 mA stimulation with the given impedance. Note that the impedance was stabilized after 20 days post implantation. (C) Left and right pulvinar nucleus LFP recording template subtracted QRS complex during stim on and off conditions. (D) Relative comparison of left and right pulvinar nucleus LFP broadband (4–75 Hz) response to the 9 different stimulation parameters during stimulation “on” only. (E) Relative comparison of left and right pulvinar nucleus LFP broadband (4–75 Hz) response to the 9 different stimulation parameters with the stimulation on response normalized by the 1 min of preceding baseline LFP response. This normalization further extracts the impact of stim on vs. stim off at each setting while showing the relative differences between settings. (D, E) The black markers represent the initial parameter space tested settings (125 Hz, 145 Hz, 165 Hz and 60us, 90us, 120us) while the green marker (165 Hz, 60us) was the selected research parameter. Stimulation current amplitude, frequency and pulse width total electrical energy delivered (TEED) was kept constant across settings when adjusting frequency and pulse width.

DBS therapy was initially programmed using standard clinical stimulation parameters (145 Hz, 90 μs) 1 day following implantation [16]. Nine different stimulation parameters were selected around the clinical setting (125 Hz, 145 Hz, 165 Hz, and 60 μs, 90 μs, and 120 μs) to capture an initial pulvinar local field potential (LFP) response to different stimulation parameters. As increasing broadband LFP power in the thalamus has been shown to correlate with seizure onset and duration [16], we compared broadband (4–75 Hz) pulvinar thalamic activity across contacts and settings to minimize broadband pulvinar LFP power bilaterally. Broadband activity was minimized using deep pulvinar contacts with continuous stimulation at 145 Hz frequency, 90 microsec pulse width, and amplitude was gradually increased to 5.9 mA over 4 days (charge density (8.85 μC/cm^2^) below 30 μC/cm^2^ limit). Figure 3 shows the change in electrode contact impedance over time. Electrode impedance stabilization occurred around 12 days post implant.

**FIGURE 4:**
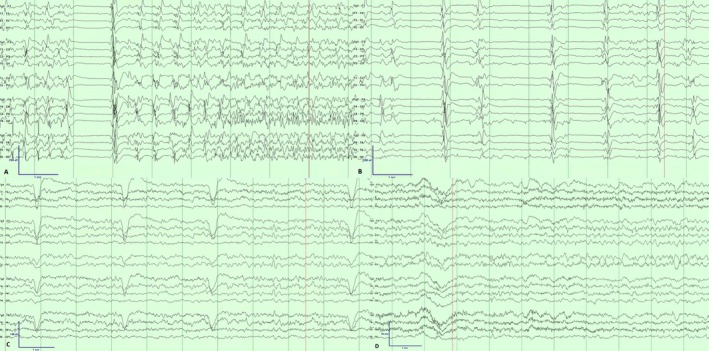
EEG on Day 12 of hospitalization demonstrating (A) bilateral seizure onset with right‐sided predominance and (B) generalized interictal discharges, more pronounced in the right posterior quadrant. Sustained seizure control was achieved by Day 8 post‐DBS implantation (Day 87 of hospitalization) (C). Later in the hospitalization during phenobarbital weaning, occasional breakthrough seizures with varying localizations were seen, such as this one recorded on Day 130 with left parasagittal onset (D).

The patient's seizures stopped on post‐op Day 8, with EEG showing ongoing generalized, bilateral independent and lateralized epileptiform discharges (Figure 4). The DBS settings were subsequently changed to continuous stimulation at 6.8 mA, 165 Hz, 90 microsecond pulse width, as broadband pulvinar LFP activity was further lowered using these settings.

The patient underwent tracheostomy placement and remained on five ASMs including clobazam, levetiracetam, lacosamide, perampanel, and phenobarbital. During the inpatient ASM weaning process, phenobarbital was gradually reduced, which led to some breakthrough subclinical seizures. He was discharged to a long‐term acute care hospital after stable seizure control was achieved for ventilator weaning after a 194‐day inpatient hospital stay. Clinically, he is now alert, interactive, and continues to improve with aggressive rehabilitation, though he remains below his cognitive and functional baseline.

## 4 Discussion

Managing NORSE requires a complex regimen of ASMs, anesthetics, and immunomodulation. In our case, control of super refractory status epilepticus was ultimately achieved with the addition of continuous stimulation targeting the pulvinar thalami.

The evidence for neuromodulation in status epilepticus in the literature is relatively sparse. Currently, most reported cases have targeted the thalamic ANT and CMT for refractory status epilepticus [8, 9, 10, 11, 12, 13]. The pulvinar nucleus is highly interconnected with the posterior parietal and occipital cortices, the cingulate gyrus, and mesial temporal regions [17]. Due to these widespread connections, it has attracted considerable interest as a potential target for neuromodulation in the treatment of epilepsy. Prior studies have demonstrated ambulatory seizure monitoring using spectral fingerprinting (12.15–17.15 Hz) through Medtronic Percept DBS electrodes implanted bilaterally in the medial pulvinar [18]. However, there are no reported cases of pulvinar DBS for the acute treatment of NORSE. One retrospective chart review of three patients in whom the pulvinar was targeted with responsive neurostimulation (RNS) achieved > 50% seizure reduction in refractory focal posterior quadrant epilepsy at 1‐year follow‐up [19]. Chronic pulvinar (PuM) stimulation has also been investigated as a therapeutic strategy for intractable temporal lobe and posterior quadrant epilepsies [18, 20]. Lastly, a systematic review summarized the five available studies that utilized transient and chronic pulvinar stimulation for epilepsy, with response rates ranging from 62.5% to 80%, with some patients experiencing > 90% seizure reduction with chronic stimulation [21]. In our case, multiple target thalamic regions were considered during surgical planning, and the pulvinar was selected after extensive multidisciplinary discussion that reached a consensus based on seizure localization to the posterior quadrant.

The patient's presentation is most consistent with NORSE. Although this patient meets FIRES criteria, the absence of identifiable etiologies, stable baseline function, unremarkable imaging and genetics support classification as NORSE. As is frequently the case for NORSE patients, establishing causal relationships between a singular treatment and clinical improvement is difficult due to the high complexity of management and differences in individual patient characteristics. For our patient, the reduction in seizure burden occurred alongside multiple ASM adjustments, allopregnanolone trial, and extensive immunotherapy including intrathecal dexamethasone in the week prior to DBS implantation. Additionally, there is the question of whether the super refractory status epilepticus would have eventually “burned out” given time, and serial imaging obtained periodically during the patient's admission did reveal global cerebral atrophy, most pronounced in the bilateral hippocampi, which is a well‐described sequela of prolonged status epilepticus and portends poor functional outcomes [22]. Although there is a clear temporal correlation between DBS activation and clinical response, we cannot attribute the patient's EEG and clinical improvement solely to DBS, as there were concurrent adjustments to the patient's ASMs. However, after DBS activation, there was a lasting reduction in seizure burden enabling the clinical team to significantly reduce severely sedating ASM doses without recurrence of status epilepticus, demonstrating sustained efficacy.

NORSE is challenging to treat, and literature on neuromodulation in status epilepticus remains limited. This is the first reported case documenting the use of DBS targeting the pulvinar for acute NORSE, where cessation of status epilepticus was achieved through continuous pulvinar stimulation after implantation, combined with conventional therapies. This case highlights the potential utility of tailoring thalamic neuromodulatory targets, such as the pulvinar, to the individual's epileptogenic network—particularly in posterior onset—while acknowledging the established roles of ANT and CMT in other localizations.

## Author Contributions

The authors take full responsibility for this article.

## Funding

The authors have nothing to report.

## Ethics Statement

This case report was determined to be exempt from IRB review in accordance with institutional policies, as it does not meet the criteria for human subjects' research requiring IRB oversight. Written informed consent for publication, including the use of clinical data and images, was obtained from the patient's legal guardian.

## Conflicts of Interest

The authors declare no conflicts of interest.

## Supporting information


**Table S1:** Selected laboratory results during the hospitalization.

## Data Availability

De‐identified clinical data supporting this case report are available from the corresponding author upon reasonable request, subject to institutional and privacy regulations.
