# Supplementary material for: Refractory Status Epilepticus Treated With Bilateral Pulvinar Deep Brain Stimulation—A Case Study
Source: Ann Clin Transl Neurol. 2025 Dec 4;13(3):627–32. doi: 10.1002/acn3.70268 (PMC12968458; doi:10.1002/acn3.70268)
Supplement: Supplementary file 1 — Table S1: Selected laboratory results during the hospitalization. [file ACN3-13-627-s001.docx]

Supplemental Table 1. Selected laboratory results during the hospitalization

| **Test** | **Day** | **Result** |
| --- | --- | --- |
| **CSF** |  |  |
| Protein | 1 | 40.5 mg/dl |
| Glucose | 1 | 64 mg/dl |
| RBC | 1 | 673 |
| WBC | 1 | 2 |
| Meningitis panel (Biofire) | 1 | Negative |
| Gram stain/aerobic culture | 1 | Negative |
| West Nile IgM/IgG | 1 | Negative |
| Lyme IgM/IgG | 1 | Negative |
| VZV PCR | 1 | Negative |
| HSV 1/2 PCR | 1 | Negative |
| VDRL | 4 | Negative |
| UCSF Metagenomics Panel | 14 | Negative |
| Autoimmune encephalitis panel (Mayo ENC2) | 3 | Negative |
|  | 55 | Negative |
| Autoimmune epilepsy panel (Mayo EPC2) | 4 | Negative |
| Igg synthesis rate/index | 14 | Within normal limits |
| Oligoclonal bands | 14 | 7 (H) |
|  | 55 | 4 (H) |
|  | 65 | 13 (H) |
| Cytokines profile (Arup) | 13 | within normal limits |
|  | 55 | mildly elevated soluble IL-2 (31.6 pg/ml, ULN <= 26.8) |
|  | 65 | mildly elevated soluble IL-2 (27.6 pg/ml, ULN <= 26.8) |
| Cytokines profile (NORSE Registry) | 69 | Mildly elevated M1P1a, possibly seizure-induced, not reflective of a highly inflammatory condition |
|  |  |  |
| **Serum** |  |  |
| Autoimmune encephalitis panel (Mayo ENS2) | 3 | Negative |
| Cytokines profile (Arup) | 13 | Elevated Il-10 (8.0 pg/ml) |
| Whole Exome Sequencing and Copy Number Variation Analysis [Trio: proband, mother, father] | 44 | Negative for known pathogenic variants |
| Cytokines profile (NORSE Registry) | 69 | Mildly to moderately elevated GCSF, IL-6, IL-10, and VEGF, possibly seizure-induced, not reflective of a highly inflammatory condition |
